# The use of digital stories as a health promotion intervention: a scoping review

**DOI:** 10.1186/s12889-022-13595-x

**Published:** 2022-06-14

**Authors:** Abby M. Lohr, Jhenitza P. Raygoza Tapia, Elizabeth Salerno Valdez, Leslie C. Hassett, Aline C. Gubrium, Alice Fiddian-Green, Linda Larkey, Irene G. Sia, Mark L. Wieland

**Affiliations:** 1 Department of Community Internal Medicine, Mayo Clinic, Rochester, USA; 2 Cancer Clinical Research Office, Mayo Clinic, Scottsdale, USA; 3 Department of Community Health Education, School of Public Health and Health Sciences, University of Massachusetts, Amherst, USA; 4 Mayo Clinic Libraries, Mayo Clinic, Rochester, USA; 5 School of Nursing and Health Professions, University of San Francisco, San Francisco, USA; 6 College of Nursing and Health Innovation, Arizona State University, Tempe, USA; 7 Department of Infectious Diseases, Mayo Clinic, Rochester, USA

**Keywords:** Digital storytelling, Storytelling, Health promotion, Health disparities, Health equity, Scoping review

## Abstract

**Background:**

It is challenging to develop health promotion interventions created in collaboration with communities affected by inequities that focus beyond individual behavior change. One potential solution is interventions that use digital stories (DS).

Digital storytelling (DST) is an opportunity for reflection, connection with others, and the elevation of voices often absent from daily discourse. Consequently, public health researchers and practitioners frequently employ the DST workshop process to develop messaging that promotes health and highlights concerns in partnership with historically marginalized communities. With participants’ permission, DS can reach beyond the storytellers through behavior or attitude change interventions for health promotion among communities who share the targeted health concern. Our goal was to synthesize the literature describing interventions that use DS for health promotion to identify gaps.

**Methods:**

We conducted a scoping review. Our inclusion criteria were articles that: 1) described empirical research; 2) used DS that were developed using the StoryCenter DST method; 3) assessed an intervention that used DS to address the health promotion of viewers (individuals, families, community, and/or society) impacted by the targeted health issue 4) were written in English or Spanish.

To synthesize the results of the included studies, we mapped them to the health determinants in the National Institute of Minority Health and Health Disparities (NIMHD) research framework. We assessed the number of occurrences of each determinant described in the results of each article.

**Results:**

Ten articles met the eligibility criteria. All the included articles highlighted health equity issues. Our mapping of the articles with definitive results to the NIMHD research framework indicates that interventions that use DS addressed 17 out of 20 health determinants. All mapped interventions influenced intentions to change health behaviors (NIMHD level/domain: Individual/Behavioral), increased health literacy (Individual/Health Care System), and/or stimulated conversations that addressed community norms (Community/Sociocultural Environment).

**Conclusions:**

Interventions that use DS appear to positively affect the health promotion of participants across a range of health issues and determinants. Future research is needed in the Interpersonal, Community, and Societal levels and within the Biological, Physical/Built Environment, and Sociocultural Environment domains.

**Supplementary Information:**

The online version contains supplementary material available at 10.1186/s12889-022-13595-x.

## Background

Numerous funding agencies have called on researchers to address health inequities, or to build infrastructure where “everyone has a fair and just opportunity to be as healthy as possible” ([1] para 1). Systems-level transformation (e.g., political, institutional, economic) is needed to achieve this goal because the root causes of such inequities are systemic in their nature and function. Yet, it is challenging to develop health promotion interventions that a) enable people to increase control over and improve their health [2] b) focus beyond individual behavior change and c) are created in collaboration with the communities most affected [3]. One potential solution is interventions that use digital stories (DS).

Digital storytelling (DST) is a type of critical narrative intervention or “an asset-based, narrative, and participatory approach to promoting health and addressing social inequality” ([4] p. 1). DST is a facilitated *process* of sharing life events that grew out of community theater in the early 1990s [5]. DS – the *product* of the DST process—are short, first-person narratives documenting experiences [6]. In this manuscript, we will use the StoryCenter model of DST as outlined by Lambert: created by individuals impacted by the health promotion theme addressed (e.g., Type 2 diabetes) and includes a voiceover narration, still or moving images, special effects, and is 1–5 min in length [7, 8]. The methods used in DST workshops are drawn from *testimonio*, popular education, and participatory filmmaking practices [6]. Within the DST workshop, there are three parts: individual, group, and co-mediated processes during which participants, researchers, and facilitators co-create knowledge. DST workshops are typically conducted with groups of 8–10 participants and facilitated by two trained professionals. Hands-on activities include expressive writing and talking activities, a story circle (where participants share nascent stories with the group), script writing, voiceover recording, and digitally editing a cut of a story. A DST workshop ends with a screening of the finalized DS where participants present and reflect on their work as a group. Since its inception, DST has been utilized in a variety of settings including education, research, policy, advocacy, and health promotion.

DST and community-based public health are natural partners. The process of creating DS is an opportunity for reflection, self-expression, connection with others who have similar experiences, and the elevation of voices often absent from daily discourse [4]. The individuals impacted by the theme are central to the production of knowledge. As a result, public health researchers and practitioners frequently employ the DST process to develop culturally-centered/community-aligned messaging to promote health and highlight issues of concern in partnership with historically marginalized communities [9, 10]. With the permission of participants, the DS that result from these DST workshops can then be used to reach a larger population (outside the workshop) in behavior or attitude change interventions for health promotion among individuals who share the targeted health concern. One example comes from the Centers for Disease Control and Prevention initiative “Bring your brave,” using stories made by people at risk for hereditary breast and ovarian cancer to increase genetic screening among at-risk young women [11].

While there is limited research suggesting impacts of the DST process on the participants [10, 12–15], a growing body of literature examines attitudinal and behavioral outcomes of health interventions that use DS [16]. Several literature reviews are published on related topics [16–20], but none have specifically addressed the impact of interventions that use DS (designed for viewers not involved in a DST workshop but impacted by the targeted health issue) on health promotion. Thus, our goal was to map and synthesize the current literature describing interventions that use DS for health promotion to identify gaps for future work. Specifically, by charting the priority population, study location, setting of the DS screening, description of the intervention that used DS, study design, theory utilized, measure(s), and outcomes of the included articles, we provide a comprehensive snapshot of the current state of the field. Due to the diversity of study designs, priority populations, and health promotion issues addressed by interventions that use DS, we deemed a scoping review the most appropriate format for this work.

Additionally, we also mapped the results of the included studies to the National Institute of Minority Health and Health Disparities (NIMHD) research framework [21]. This model depicts levels (Individual, Interpersonal, Community, and Societal) on the horizontal axis and domains (Biological, Behavioral, Physical/Built Environment, Sociocultural Environment, Healthcare System) on the vertical axis that intersect to form determinants impacting health equity. Additionally, the framework includes a vertical, bidirectional life course perspective arrow signifying the importance of considering early adverse events, chronic and cumulative exposures, and transgenerational transmission of risk and resilience when assessing the domains of influence [21]. Through this process, we demonstrate how interventions that use DS have addressed health determinants as well as future directions for DST researchers and practitioners.

## Methods

The scoping review was drafted using the Preferred Reporting Items for Systematic Reviews and Meta-analysis Extension for Scoping Reviews (PRISMA-ScR) [22] and the Johana Briggs Institute Manual for Evidence Synthesis [23] (see Additional file 1: PRISMA-ScR Checklist). The research team wrote and registered the scoping review protocol prospectively with Open Science Framework [24] on September 29, 2021 (Registration https://doi.org/10.17605/OSF.IO/WZD4G).

### Eligibility criteria

To capture all examples, we included articles that prioritized any clearly defined population (e.g., people living with HIV instead of the general public) and health promotion topic. We did not limit our search using a specific time frame. To draw information for description and synthesis among a DS-delineated set of studies, we used the following eligibility criteria: peer-reviewed journal articles that: 1) described empirical research; 2) used DS that were developed through StoryCenter’s DST workshop process as the health promotion intervention; 3) assessed the effects of an intervention that used DS on the health promotion of viewers impacted by the targeted health issue; 4) were written in English or Spanish (both reviewers are bilingual). Additionally, to remain focused on clearly defined populations, we excluded articles describing the use of DS in formal educational settings such as medical school or for exclusively therapeutic contexts (e.g., to reduce anxiety prior to a medical procedure without the goal of changing health behavior).

### Information sources

An experienced librarian (LCH) designed the search strategy with input from the coauthors. We conducted a comprehensive search of several databases, limited to English language, on September 21, 2021. We re-ran the search on November 17, 2021 to update the original search and to include both English and Spanish language articles. Although we registered our protocol after we conducted our original search, we did not begin screening articles until the protocol had been approved by Open Science Framework on October 2, 2021. The databases we searched included: Ovid MEDLINE(R) and Epub Ahead of Print, In-Process & Other Non-Indexed Citations and Daily, Ovid Embase, Ovid Cochrane Central Register of Controlled Trials, Ovid Cochrane Database of Systematic Reviews, APA PsycInfo, CINAHL, and Scopus. Additionally, we hand searched the reference lists of all included articles as well as other literature reviews published in this topic area to ensure we identified all relevant articles. We present an example of the electronic search strategy for Ovid databases in Table 1. The complete search strategy for all databases is available in Additional file 2.

**Table 1:** Example search strategy for Ovid databases^a^ for scoping review on the use of digital stories as a health promotion intervention

#	Search Text
1	(digital or visual) adj1 (story or stories or storytelling or "participatory research").ti,ab,hw,kw.^b^
2	remove duplicates from 1
3	limit 2 to (english or spanish) [Limit not valid in CDSR; records were retained]

^a^Database(s): Ovid MEDLINE(R) 1946 to Present and Epub Ahead of Print, In-Process & Other Non-Indexed Citations and Ovid MEDLINE(R) Daily, APA PsycInfo 1987 to November Week 2 2021, EBM Reviews—Cochrane Central Register of Controlled Trials October 2021, EBM Reviews—Cochrane Database of Systematic Reviews 2005 to November 11, 2021, Embase 1974 to 2021 November 16

^b^*ti* title, *ab* abstract, *hw* subject heading word, *kw* keyword heading

### Selection of sources of evidence

We imported the search results from the databases into EndNote reference software and then into Covidence [25], a platform for screening articles in literature reviews. Two independent reviewers (AML and JPRT) screened titles and abstracts for eligibility. The two reviewers then read the full text of the articles meeting the inclusion criteria.

We contacted four authors via email to attain a full text copy of one article and to determine whether the DS in three additional articles were created through the DST process as defined by StoryCenter. One author responded and confirmed that she used StoryCenter’s DST process. In the other two cases, a third reviewer (MLW) joined the discussion and together the reviewers reassessed the evidence using only the information presented in the respective articles to make a final decision.

### Data charting process

The two reviewers independently charted the data from included studies using a piloted form (AML and JPRT). We included the following information in the data chart: author, year of publication, country where the study was conducted, setting of the DS screening (e.g., clinical), description of the intervention that used DS, priority population and sample size, study design, theory utilized, measure(s), and outcomes. We wrote a scoping review, meaning we included all existing articles that met our inclusion criteria regardless of methodological quality. Thus, we did not conduct a quality assessment of the included articles [22].

### Synthesis of results

Three authors (AML, JPRT, and MLW) synthesized definitive study results (statistically significant quantitative results and/or qualitative findings) by mapping them to the determinants described in the National Institute of Minority Health and Health Disparities (NIMHD) research framework [21]. To map findings, we examined the number of occurrences of each determinant described in the results of each included article using the following process. The first author re-read the results and discussion section of each article to understand the authors’ findings in context. Next, she adapted the NIMHD framework chart by inserting author names and years to document which determinants were addressed by each article. For example, Chia-Chen Chen et al., found that after viewing a DS created by Vietnamese mothers, other mothers changed their attitudes and beliefs toward human papilloma virus (HPV) vaccination and intent to vaccinate their adolescent children [26]. The first author categorized these results into three determinants:

1. Societal / Biological: because the intervention addressed immunization;
2. Individual / Behavioral: because the intervention addressed behavior change around HPV vaccination; and
3. Individual / Health Care System: because the intervention addressed health literacy around HPV vaccination.

The second author repeated the same process and we discussed disagreements. The last author clarified questions around the biological domain of influence.

The application of the NIMHD research framework is appropriate in this scoping review because most DST projects prioritize historically marginalized individuals who experience health disparities [14, 16, 27–32]. By applying the NIMHD research framework to the included studies, we sought to use a standardized model to summarize and integrate our findings and describe the strengths and opportunities in the current research. In this process, we excluded results that described the likeability, feasibility, or relevance of the DS used because in this manuscript we were interested in the impact of interventions that used DS on health promotion.

## Results

### Selection and characteristics of sources of evidence

**Fig. 1:**
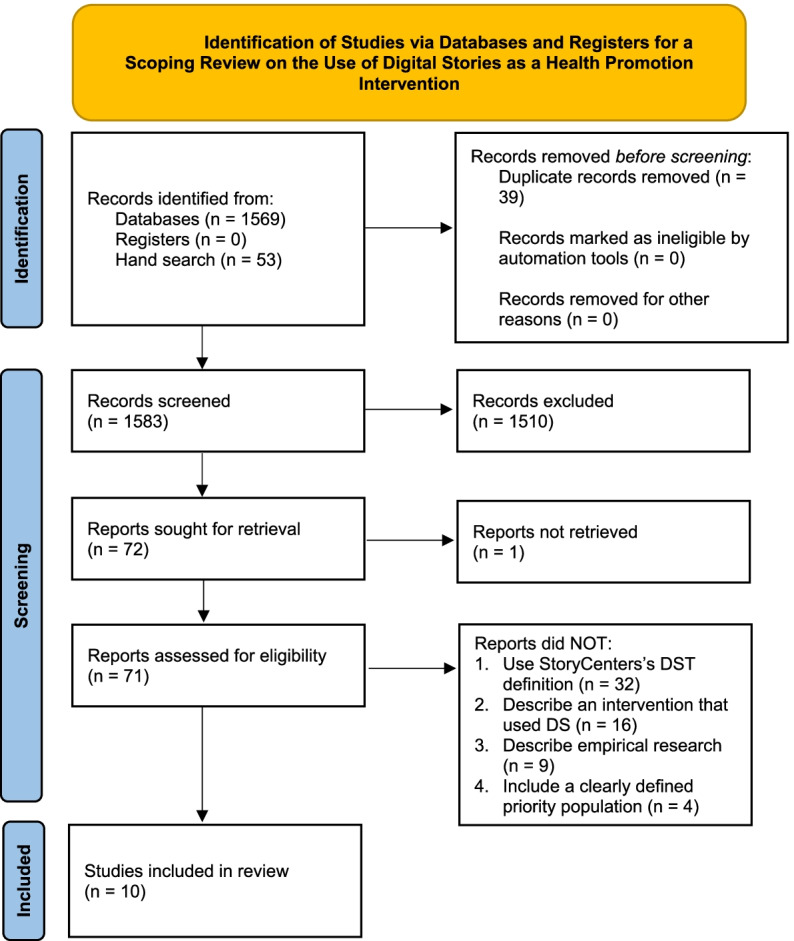
Identification of Studies via Databases and Registers for a Scoping Review on the Use of Digital Stories as a Health Promotion Intervention

We identified 1569 records from the database and 53 records through hand searching (Fig. 1). We removed duplicates and reviewed the title and/or abstract for the remaining 1583 articles to determine eligibility for full text review. The two independent reviewers screened 71 full texts. Ten articles met the eligibility criteria. In the case of Willis et al. and Flicker et al., the authors incorporated the results of assessments of the DS creators and viewers within the same article. Here, we only report results pertaining to the DS viewers. Additionally, it is noteworthy that the authors of the Carlson et al. and Wieland et al. articles are part of the same research team and thus both articles describe the same intervention that assessed a DS in different ways. Finally, we also noted that the DST creators and viewers knew each other (which is often the case in community-based interventions) in three articles: Willis et al., Cueva et al. 2015, and Jernigan V et al.

All the included articles highlighted health equity issues. The researchers administered their interventions in the United States [33–37], the United Kingdom [38], Canada [39], South Africa [40], and Zimbabwe [41]. They screened the DS in clinical [33, 34, 37, 40], community [26, 35, 36, 39, 41] or school-based [38] settings. In most studies, viewers had only one method of watching the DS: in a group setting [26, 33, 35, 36, 38, 39, 41], a clinic waiting room [40], or individually in a private room at a clinic [37]. In contrast, in the Cueva et al., study conducted in 2015, viewers had the option to watch online, at the health clinic, at home, at a community showing, at a local business, or at work [34]. The authors prioritized several groups including: Latino adults [33, 37], young people living in a low-income setting in South Wales [38], Vietnamese mothers [26], Indigenous youth or adults [34–36, 39], individuals living in rural South Africa [40], Somali adults [37], and caregivers to youth living with HIV [41]. The sample size of DS viewers ranged from 10 [26] to 860 people [40]. The researchers addressed a variety of health promotion topics: Type 2 Diabetes (T2D) [33, 37], binge drinking [38], HPV vaccination [26], cancer awareness and education [34, 35], HIV [39–41], and food insecurity [36].

To guide their work, many of the researchers used the Theory of Culture-Centric Narratives in Health Promotion [26, 34, 35, 42] and two authors combined this theory with the Social Cognitive Theory [33, 37, 43]. Other authors used the Theory of Planned Behavior [38, 44], Indigenous epistemology and ontology [34], the Bioecological Model of Human Development [39, 45], the Tool for Health and Resilience in Vulnerable Environments (THRIVE) Policy and Engagement Framework [36, 46], Freire’s Theoretical Framework of Empowerment [40, 47], and social constructionism in the context of narrative therapy [41, 48].The researchers also utilized several different study designs to assess the impact of the intervention that used DS including quasi-experimental study [26, 33, 38, 40], case study [34–36, 39, 41], and cross-sectional study [37]. Within these designs, the authors employed diverse methods: five qualitative [34–36, 39, 41], two quantitative [26, 38], and three mixed methods [33, 37, 40]. In five cases, the researchers included focus group discussions [33, 35, 36, 40, 41] and one research group analyzed audio-recordings of audience reflections [39] to gather data from conversations that occurred after the DS viewing (Table 2).

**Table 2:** Data extraction chart: studies included in the scoping review on the use of digital stories as a health promotion intervention

**Author(s), Year**	**Country**	**Setting of Digital Story Screening**	**Description of the Digital Storytelling Intervention**^a^	**Study Design**	**Theory Used**	**Measure(s)**	**Outcomes**
Carlson et al. (2020)^b^ [33]	U.S.A	Clinical	Creators: Latino, Spanish-speaking patients with Type 2 Diabetes (T2D) Viewers: Latino adults diagnosed with T2D living in rural areas (*n* = 23) Intervention: Group viewing of digital stories followed by discussion	Quasi-experimental	Social Cognitive Theory and Culture-Centric Narratives in Health Promotion	Qualitative: Observational notes and audio recordings of focus groups Quantitative: Pre/Post intervention surveys	Qualitative: Sessions rated as highly acceptable, interesting, and useful; Improvements in confidence, motivation, and behavioral intentions for T2D self-management, facilitated discussions may add value to viewing DS Quantitative: Statistically significant findings reflected in measurements of both ‘motivation for’ (*p* < 0.01) and ‘confidence in’ T2D self-management (*p* = 0.02)
Coleman, Ramm, and Cooke (2010) [38]	United Kingdom	School	Creators: Young people (ages not specified) created digital stories to address the consequences of binge drinking Viewers: Young people (14–16 years old) who drink alcohol (*n* = 89 participants matched between timepoints two and three included in inferential analysis) Intervention: Group viewing of digital stories followed by discussion and questionnaires at three timepoints	Quasi-experimental	Theory of Planned Behavior	Quantitative: Questionnaires one-month prior to the intervention, immediately after viewing the intervention, and six months after the intervention	Quantitative: Positive effect on knowledge for the intervention sample (F = 3.35; p = 0.07); Intervention participants got drunk fewer times in the last week compared to controls (F = 1.90; *p* = 0.07)
Chia-Chen Chen, Wonsun, and Larkey (2019) [26]	U.S.A	Community	Creators: Vietnamese American mothers of children vaccinated against HPV Viewers: Vietnamese American mothers of at least one unvaccinated child between the ages of 11–17 years old (*n* = 10) Intervention: Group viewing of digital stories followed by surveys	Quasi-experimental	Culture-Centric Narratives in Health Promotion	Quantitative: Pre/Post intervention surveys	Quantitative: Statistically significant findings in the knowledge (effect size = 1.0; *p* = 0.03) and attitudes (effect size = 0.8; *p* = 0.05) around HPV vaccination. The intervention was determined feasible and acceptable to participants. All participants reported their intent to vaccinate their children
Cueva, Kuhnley, Revels, Schoenberg, and Dignan (2015) [34]	U.S.A	Clinical	Creators: Community Health Aide/Practitioners (CHA/P) created short 2–3-min movies on the topics of wellness, cancer risk reduction and prevention, and screening for early detection and treatment Viewers: Rural community members (*n* = 15) Intervention: Community members watched the DS online, at the health clinic, at home, at a community showing, at a local business, or at work. They were invited to participate in a telephone interview 1–5 months after watching the DS	Case Study	Culture-Centric Narratives in Health Promotion and Indigenous Epistemology and Ontology	Qualitative: Post intervention interview	Qualitative: Digital stories reported as an “emotionally engaging” approach, a starting place for discussions on inner reflection, insight, and cancer prevention. Emphasis on indigenous epistemology and ontology stemming from prioritized relationships and interconnectedness
Cueva et al. (2016) [35]	U.S.A	Community	Creators: Community Health Workers (CHWs) created digital stories on tobacco cessation, colon and breast screening, treatment, and early detection of cancer Viewers: Alaska Native community members. (*n* = 29) Intervention: Group viewing of digital stories followed by questionnaire and discussion	Case study	None listed	Qualitative: Open-ended questionnaire and focus groups with written and verbal comments	Qualitative: Participants described digital stories as being culturally respectful, engaging, informational, inspiring, and motivational
Flicker et al. (2020) [39]	Canada	Community	Creators: Indigenous youth who participated in digital storytelling workshops on HIV activism Viewers: Members from the youths’ community and internationally (number of viewers not listed) Intervention: Youth hosted group screenings in their communities followed by discussions	Case study	Bioecological Model of Human Development	Qualitative: Semi-structured qualitative interviews with youth creators and discussion with audience members	Qualitative: the impact of digital stories was seen at the macro (policy), meso (family, peers, and community), and micro (youth) levels. The digital stories sparked conversations in the community about HIV prevention and care. Community support spread through kinship networks
Jernigan, Salvatore, Styne, and Winkleby (2012) [36]	U.S.A	Community	Creators: Native American community leaders Viewers: Community members (*n* = 40) Intervention: Group viewing of DS followed by focus groups	Case study	Tool for Health and Resilience in Vulnerable Environments (THRIVE) Policy Engagement Framework	Qualitative: Focus Groups	Qualitative: Community members identified racial injustice and both physical and financial barriers to accessing healthy and culturally appropriate foods as areas of greatest importance. This outcome resulted in creation of local policies to reduce identified barriers
Treffry-Goatley, Lessells, Moletsane, de Oliveira, and Gaede (2018) [40]	South Africa	Clinical	Creators: Community members recruited from primary healthcare programs discussing HIV and Adherence to Antiretroviral Therapy (ART) Viewers: Digital stories were disseminated to 7 local public health clinics for patients’ viewing. Respondents before screening (*n* = 852), Respondents after screening (*n* = 860), Participants from the general public, healthcare workers, and Community Advisory Board (*n* = 65) Intervention: Viewed DS in waiting room followed by survey and discussion	Quasi-experimental	Freire’s Theoretical Framework of Empowerment	Qualitative: Focus groups, and observation of individuals watching digital stories Quantitative: Surveys	Qualitative: Focus groups revealed that DS are an effective way to engage people and stimulate discussion around HIV and its treatment Quantitative: Descriptive statistics demonstrated no difference in knowledge or understanding of HIV or ART between intervention and control participants
Wieland et al. (2017)^b^ [37]	U.S.A	Clinical	Creators ^c^: Latino and Somali storytellers completed a digital storytelling workshop onT2D self-management Viewers: Latino and Somali patients (*n* = 25) Intervention: Individual viewing of digital stories followed by face-to-face interviews and blood glucose measurement	Cross-sectional structured interviews; Cohort Study	Narrative Theory and Social Cognitive Theory	Qualitative: interviews to assess intervention acceptability, interest level, usefulness, self-rated confidence, and motivation for managing T2D Quantitative: measures of A1C for intervention feasibility and preliminary evidence	Qualitative: High acceptability, stated to be interesting, and useful. Reported a range of main messages coinciding with intention to change T2D related behavior, more confident about managing T2D after watching the video, and plans to share video Quantitative: hemoglobin A1C change was statistically significant among Latino participants (-1.5% [-17 mmol/mol] change from baseline; *p* = 0.03) but not Somali participants (-0.4% [-4 mmol/mol] change from baseline; *p* = 0.36)
Willis et al. (2014) [41]	Zimbabwe	Community	Creators: Young people (18–22 years old) from (HIV) Africaid Zvandiri programme Viewers: Primary caregivers of the creators (*n* = 12) Intervention: Group viewing of digital stories followed by discussion	Case study	Social Constructionism in the context of narrative therapy	Qualitative: One focus group with caregivers	Qualitative: Caregivers stated that after watching the DS they had a better understanding of their children and that the intervention helped share memories of people who had died and in some cases helped individuals accept their own HIV status

^a^Viewers were not creators of the intervention

^b^The Carlson et. al. and Wieland et. al. articles come from the same research group and thus use the same digital stories

^c^Information extracted from Njeru J.W., et al. (2015) [49]

The authors reported a variety of outcomes. Viewers described the DS as acceptable [26, 33, 37], useful, interesting [33, 37], feasible [26], engaging [34, 35], culturally respectful, informational, inspiring, and motivational [35]. Three studies had statistically significant findings around motivation for and confidence in T2D self-management [33], change in blood glucose [37], or knowledge and attitudes around HPV vaccination [26]. These three articles also reported improvements in confidence, motivation, and/or behavior change intentions around T2D self-management [33, 37] or HPV vaccination [26]. In contrast, while Coleman et al., found that their DS had a positive effect on knowledge and that intervention participants got drunk fewer times in the last week compared to controls, these findings were not statistically significant [38]. Four of the articles that employed qualitative methods emphasized that DS are an effective way to engage people and stimulate discussions on inner reflection, insight, shared memories, or health promotion [34, 39–41]. Two articles described how the DS intervention resulted in social change. Flicker et al., reported that the impact of the DS was seen at the macro (policy), meso (family, peers, and community), and micro (youth) levels. The DS reached policy makers and challenged conventional public health messaging around HIV, instead situating it within an Indigenous conception of health [39]. Jernigan V et al., reported that community members identified racial injustice and both physical and financial barriers to accessing healthy and culturally appropriate foods. This outcome resulted in the creation of local policies to reduce identified barriers [36].

### Mapping of studies

By mapping the definitive results to the NIMHD research framework, we learned that two articles addressed all the levels of influence [39, 40] and six articles addressed three of five domains of influence [26, 34–36, 40, 41]. The included articles primarily focused on the Individual level of influence (15 occurrences) and the Behavioral domain of influence (14 occurrences). Consequently, the most addressed determinant (level/domain cross section) was Individual/Behavioral with seven occurrences which included interventions that addressed health behaviors or coping strategies. The second most addressed determinants with five occurrences each were 1) Individual/Healthcare System—interventions that improved health literacy; and 2) Community/Sociocultural Environment – interventions that addressed community norms and/or local structural discrimination. The latter was frequently addressed when study teams, especially those led by or collaborating with Indigenous peoples, held community forums to screen DS and discuss community members’ reflections.

**Fig. 2:**
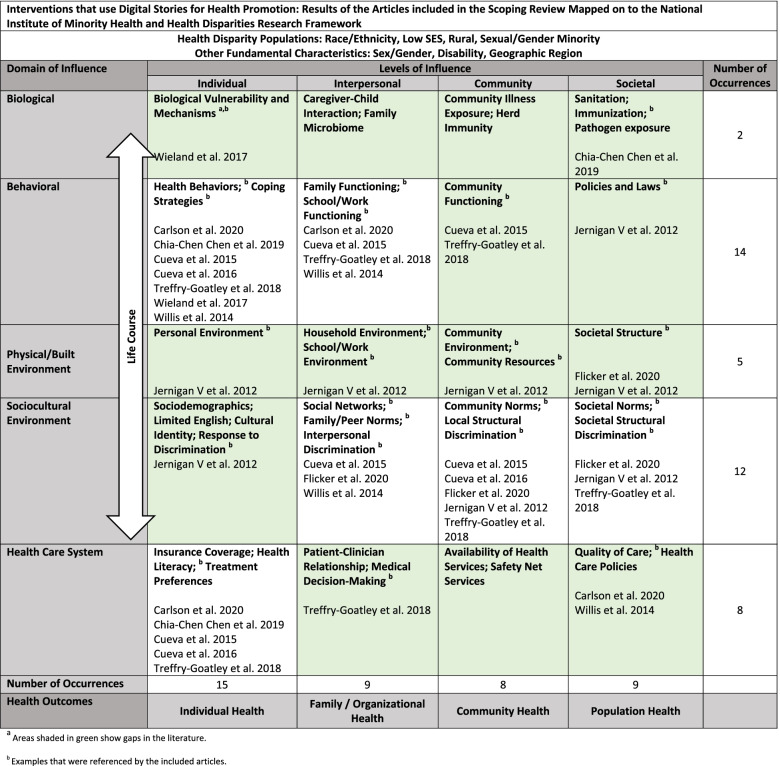
Interventions that use Digital Stories for Health Promotion: Results of the Articles included in the Scoping Review Mapped on to the National Institute of Minority Health and Health Disparities Research Framework

Fewer articles had results pertaining to the Interpersonal and Societal levels of influence. For example, four determinants were addressed by two to three articles including: Interpersonal/Behavioral (e.g., family functioning); Interpersonal/Sociocultural Environment (e.g., social networks); Societal/Physical, Built Environment (e.g., societal structure); and Societal/Sociocultural Environment (e.g., societal norms). Seven determinants were addressed by only one article, most of which were either at the Societal level or in the Physical, Built Environment domain. Only two articles each addressed the Biological domain and Physical/Built Environment domains. The Jernigan V et al. article attended to the most determinants (eight) [36] while the Wieland et al. article addressed the fewest (two) [37]. Three determinants were not addressed by any of the included articles: Interpersonal/Biological (e.g., family microbiome), Community/Biological (e.g., herd immunity), and Community/Health Care System (e.g., safety net services) (Fig. 2). None of the included articles specifically discussed the life course perspective.

## Discussion

Our aim in this scoping review was to synthesize the literature on the impact of interventions that used DS for health promotion. We found ten articles that met our eligibility criteria, which we then synthesized using the NIMHD research framework.

Our mapping of the articles with definitive results to the NIMHD research framework indicates that interventions that use DS addressed 17 out of 20 health determinants. All mapped interventions influenced intentions to change health behaviors (Individual/Behavioral), increased health literacy (Individual/Health Care System), and/or stimulated conversations that addressed community norms (Community/Sociocultural Environment). The three determinants not addressed by any articles (Interpersonal/Biological, Community/Biological, and Community/Health Care System), as well as the fourteen determinants addressed by only one or two articles, highlight the gaps in the literature (shaded green area in Fig. 2).

Additionally, we observed that none of the included articles measured actual behavior change resulting from interventions that used DS. All the articles addressing the Individual/Behavioral determinant described participants’ intentions to change behavior. Wieland et al. demonstrated biological changes because their intervention resulted in a reduction in blood sugar levels among Latino participants with Type 2 Diabetes [37]. However, the authors did not measure the specific behavior(s) that caused this change. Thus, because intentions do not always result in action, future research is needed to measure actual behavior change resulting from interventions that use DS to improve our understanding of this possible causal pathway. Gathering such data gets more difficult the further up the collective target ladder the intervention addresses (e.g., using DS in community settings to promote increases in colorectal cancer screening may require assessing population level outcomes of age-defined population denominators). The value of obtaining community or population level data, however, will increase the validity of evidence gathered for understanding the impact of interventions that use DS.

Despite these shortcomings, interventions that use DS could be or have been developed that measure actual behavior change and/or attend to the three unaddressed determinants. It is possible that such projects have been led by community-based organizations outside of academia or are still in process and therefore not yet documented in the peer reviewed literature. For example, the CDC’s Bring Your Brave (BYB) campaign focuses on the Interpersonal/Biological determinant. In BYB, women younger than age 45 who are at risk for hereditary breast and ovarian cancer created DS about cancer prevention, risk, family history, and survivorship. The CDC compiled these stories to create an online public health campaign to encourage women to have family conversations about cancer risk and to talk to their healthcare providers about screening [11]. Researchers could measure actual behavior change by assessing the number of individuals who seek genetic counseling and testing postintervention. For the Community/Biological determinant, StoryCenter is currently (as of this writing) offering free story sharing and writing workshops for persons of color identifying as queer or trans to reflect on their experiences during the COVID-19 pandemic. With participants’ permission, these DS could be screened for LGBTQIA+ populations in combination with a post-viewing community discussion to promote vaccination. Behavior change could be measured by the number of individuals vaccinated. To address the Community/Health Care System determinant, individuals who struggle to access health care services could create DS that reflect their experiences. These DS could be screened for the public and local policy makers followed by a discussion to generate ideas around and support for the development of safety net services. Evaluators could measure safety net service user health-related behaviors over time (e.g., smoking cessation).

This synthesis of results, exploration of interventions that use DS documented outside the literature, and consideration of possibilities suggests that interventions that use DS have the potential to address all the health determinants in the NIMHD research framework and thus health equity at all levels and domains. While the article by Jernigan V et al. addressed eight determinants, most articles only addressed two to three determinants. Based on our synthesis of interventions that use DS using the NIMHD framework, researchers and funding agencies may be underutilizing DS as a way to promote health equity. In the future, investigators and funders should consider the versatility and incredible adaptability of DS to address a wide range of health determinants across the levels and domains of influence.

To achieve this aim, we present two suggestions gathered from the articles included in this scoping review. First, we urge individuals who are not from Indigenous communities to listen to, learn from, and give credit to Indigenous wisdom around community engagement to increase the impact of interventions that use DS at the community level. Most of the articles that attended to the Sociocultural domain described work with and by Indigenous researchers and participants. This reflects Indigenous understandings of health, which directly link individual and community well-being [39] through Indigenous epistemologies and ontologies [34]. This finding indicates that, while other authors demonstrated that interventions that used DS impacted individual intentions to change behavior, these interventions are potentially more far reaching within a community context. Hammond et al., who conducted a scoping review on arts-based research methods (ABMs) with Indigenous peoples, had similar findings. The authors found that ABMs, including DST, have the potential to mobilize Indigenous communities and could be used toward building an Indigenous research agenda that breaks away from the colonial cycle of being researched. The authors suggest this can be achieved by researching back referring to Smith’s idea of disrupting the Western paradigm developed by colonists that researchers have an ‘objective’ or ‘neutral’ gaze by replacing racist, ethnocentric, exploitative practices with methods that are more respectful, ethical, compassionate, and useful [50]. By researching back, Hammond et al., found that the outcomes of the ABM projects often resulted in increased community readiness and capacity for implementing positive change [18].

Second, we encourage individuals employing interventions that use DS to consider conducting post-viewing discussions with the storytellers present (if feasible and appropriate). These discussions can support viewers in 1) processing the DS and gaining a deeper understanding of the issue 2) defining relevant issues; 3) realizing common strengths; and 4) discussing solutions and advocacy strategies as a group to address a wide range of health determinants through collective action. In the six cases where researchers conducted post-viewing discussions after the DS screening, the authors emphasized the benefits of a group conversation to assist viewers in contextualizing, reflecting on, and processing the DS together [33, 35, 36, 39–41]. For example, Carlson et al. stated that participants in their study preferred the combined format as it likely contributed to motivation for behavior change, served as a forum to learn T2D disease management skills, and provided social support [33]. Similarly, Treffry-Goatley et al. highlighted how the DS sparked valuable community health dialogue even on stigmatized topics such as HIV and sex among individuals whose voices are often absent in a patriarchal community – in this case Indigenous women from a rural area [40]. Jernigan, V. et al. went a step further by asking participants to reflect on their experiences and seek solutions. In these focus groups, community members discussed social and environmental factors affecting their health as a way of identifying strategies and building support for change [36].

Post viewing discussions also allow for more in-depth conversations and improved understanding of sensitive issues or experiences, especially if the storytellers are present and known by the viewers. This was illustrated by Flicker et al. who reported that many community members who attended DS screenings were willing to discuss HIV, a highly stigmatized and taboo topic, because the youth storytellers from their community led the session [39]. Similarly, Willis et al. learned that the DS screening was often the first time the storytellers (adolescents living with HIV) had openly and directly shared their experiences with their caregivers. In response, the caregivers stated that these new insights would improve communication in their family [41].

Administering an intervention that uses DS followed by a discussion in a clinical setting, however, may be challenging due to logistics and privacy concerns. A possible solution may be planning small group DS screenings and discussions with two to three patients experiencing the same diagnosis. Alternatively, as suggested by Carlson et al., clinics could hold DS screenings and facilitated discussions for patients and family members to help caregivers better understand and support their loved one’s experiences [33]. More research is needed to understand how to implement post viewing discussions as part of interventions that use DS in clinical settings.

### Implications for policy or practice

Because interventions that use DS have the potential to address multiple health determinants in an accessible, culturally sensitive way with communities impacted by health inequities, this type of intervention may inform more equitable healthcare policy. Furthermore, interventions that use DS for individual behavior or attitude change can be easily scaled and incorporated into a menu of educational opportunities for patients because it is a low-cost, portable, quick intervention that will require minimal extra effort from healthcare providers. Although addressing interpersonal, community, or societal change may require more resources, this scoping review indicates that these are worthy investments because the impacts of interventions that use DS at these levels may be broader.

### Strengths and limitations

This scoping review identified a wealth of examples of how interventions that used DS impact health promotion. To date, review studies on DST have focused on the use of DST as a health intervention in research [16, 17]. Here we concentrated on the application of interventions that use DS for health promotion. Additionally, to our knowledge we are the first to focus on DS as a knowledge translation intervention outside the DST workshop.

A limitation of the literature was that all examples used one of only three study designs: case study, quasi-experimental study, and cross-sectional study. Thus, all conclusions were drawn based on associations. To strengthen our understanding of the effects of interventions that used DS, it is critical that future researchers expand this work by using randomized and longitudinal study designs to measure causality. Additionally, while two included articles addressed all the levels of influence, none addressed more than three (of five) domains of influence. Together, the domains of influence (Biological, Behavioral, Physical/Built Environment, Sociocultural Environment, Health Care System) represent the life course perspective. More research is needed on how to develop interventions that use DS to address issues across the life course perspective.

We limited the interventions examined to DS-based messaging drawn directly from the DST method of building stories. This was done to provide a standard that assured that the voices of members of the community were represented. There are other ways that storytelling messages are incorporated into health promoting interventions (even some of these producing some type of DS, including stories and messages drawn from community members) [51]. Since these types of interventions often do not have the same level of standardized methods for assuring the cultural and/or community voice is incorporated (such as when community members or advisory boards are tapped to compile a single fictional story told in person or via DS [52]) we did not include them in this review. As such, a body of storytelling-based research that may meet the criteria of being sourced within-culture but did not subscribe to the StoryCenter version of story production, is missed. Additionally, we limited our search to articles in English and Spanish and thus may have missed DS interventions documented in other languages.

Regarding the methods of evaluation and synthesis, another limitation is the ambiguity of the NIMHD research framework. While NIMHD provided examples for each health determinant, the authors did not include definitions. Thus, although two researchers mapped the article results to the research framework, it is possible that other investigators would have interpreted the results differently. Furthermore, one of our included articles, Coleman et al., had null results meaning not all interventions that use DS may have a significant impact on viewers. Thus, our conclusions should be interpreted with caution. Finally, we restricted the review to peer-reviewed articles. Consequently, we may have missed rich examples of interventions, such as the BYB campaign, that use DS in the grey literature. This is an important point because so often DS are shared outside of academia on websites, blogs, and social media.

## Conclusions

In this scoping review, we identified 10 examples of how interventions that used DS can impact health promotion. This promising intervention appears to positively affect the health promotion of participants across a range of diseases and public health issues. By mapping the definitive results from these articles onto the NIMHD research framework, we learned that interventions that use DS have the potential to address a wide range of health determinants. Future research is needed to investigate the impact of DS on the Interpersonal, Community, and Societal levels and within the Biological, Physical, Built Environment, and Sociocultural Environment domains.

## Supplementary Information

**Additional file 1.**

**Additional file 2.**

## Data Availability

Data sharing is not applicable to this article as no datasets were generated or analyzed during the current study. Our complete literature search strategy is available in Additional file 1.

## Abbreviations

ABM: Arts-based research methods
BYB: Bring Your Brave
CDC: Centers for Disease Control and Prevention
DS: Digital stories
DST: Digital storytelling
NIMHD: National Institute of Minority Health and Health Disparities
T2D: Type 2 diabetes
