# Supplementary material for: The use of digital stories as a health promotion intervention: a scoping review
Source: BMC Public Health. 2022 Jun 14;22:1180. doi: 10.1186/s12889-022-13595-x (PMC9192132; doi:10.1186/s12889-022-13595-x)
Supplement: Supplementary file 2 — Additional file 2. [file 12889_2022_13595_MOESM2_ESM.docx]

**Appendix B. Complete Search Strategies**

**OVID**

Database(s): **Ovid MEDLINE(R) 1946 to Present and Epub Ahead of Print, In-Process & Other Non-Indexed Citations and Ovid MEDLINE(R) Daily, APA PsycInfo**1987 to November Week 2 2021**, EBM Reviews - Cochrane Central Register of Controlled Trials**October 2021**, EBM Reviews - Cochrane Database of Systematic Reviews**2005 to November 11, 2021**, Embase**1974 to 2021 November 16
Search Strategy:

| **#** | **Searches** |
| --- | --- |
| 1 | ((digital or visual) adj1 (story or stories or storytelling or "participatory research")).ti,ab,hw,kw. |
| 2 | remove duplicates from 1 |
| 3 | limit 2 to (english or spanish) [Limit not valid in CDSR; records were retained] |

**SCOPUS**

| 1 | - TITLE ((digital or visual) W/1 (story or stories or storytelling or "participatory research")) |
| --- | --- |
| 2 | INDEX(embase) OR INDEX(medline) OR PMID(0* OR 1* OR 2* OR 3* OR 4* OR 5* OR 6* OR 7* OR 8* OR 9*) |
| 3 | 1 not 2 |
| 4 | DOCTYPE(ed) OR DOCTYPE(bk) OR DOCTYPE(er) OR DOCTYPE(no) OR DOCTYPE(sh) OR DOCTYPE(ch) |
| 5 | 3 not 4 |
| 6 | 5 and ( LIMIT-TO ( LANGUAGE , "English" ) OR LIMIT-TO ( LANGUAGE , "Spanish" ) ) |

**CINAHL**

| **#** | **Search Terms** |
| --- | --- |
| S3 | s2 Limiters - English Language or Limiters – Spanish Language |
| S2 | s1  Limiters - Exclude MEDLINE records |
| S1 | ((digital or visual) N1 (story or stories or storytelling or "participatory research")) |
